# Discovery of Sulforaphane as an Inducer of Ferroptosis in U-937 Leukemia Cells: Expanding Its Anticancer Potential

**DOI:** 10.3390/cancers14010076

**Published:** 2021-12-24

**Authors:** Giulia Greco, Michael Schnekenburger, Elena Catanzaro, Eleonora Turrini, Fabio Ferrini, Piero Sestili, Marc Diederich, Carmela Fimognari

**Affiliations:** 1 Dipartimento di Scienze per la Qualità della Vita, Alma Mater Studiorum-Università di Bologna, Corso d’Augusto 237, 47921 Rimini, Italy; giulia.greco9@unibo.it (G.G.); elena.catanzaro2@unibo.it (E.C.); eleonora.turrini@unibo.it (E.T.); 2 Laboratoire de Biologie Moléculaire et Cellulaire du Cancer, Hôpital Kirchberg, 9 Rue Edward Steichen, L-2540 Luxembourg, Luxembourg; michael.schnekenburger@lbmcc.lu; 3 Dipartimento di Scienze Biomolecolari, Università degli Studi di Urbino Carlo Bo, 61029 Urbino, Italy; f.ferrini2@campus.uniurb.it (F.F.); piero.sestili@uniurb.it (P.S.); 4 Department of Pharmacy, College of Pharmacy, Seoul National University, 1 Gwanak-ro, Gwanak-gu, Seoul 08826, Korea; marcdiederich@snu.ac.kr

**Keywords:** natural products, sulforaphane, anticancer activity, non-canonical cell death, ferroptosis, necroptosis, apoptosis

## Abstract

**Simple Summary:**

Ferroptosis and necroptosis are two non-apoptotic programmed cell death pathways with increasing therapeutic potential. The isothiocyanate sulforaphane (SFN) is a well-known naturally derived anticancer compound with remarkable pro-apoptotic activity. Its ability to promote non-apoptotic cell death mechanisms remains poorly investigated. This work discovered that SFN activates apoptosis and ferroptosis dose-dependently in acute myeloid leukemia cells. At lower concentrations, SFN induces caspase-dependent apoptosis. At higher concentrations, ferroptosis is activated and accompanied by the depletion of intracellular glutathione (GSH) and decreased GSH peroxidase 4 protein expression levels. Necroptosis, instead, is not involved in SFN-induced cell death. Considering that cancer cells resist pro-apoptotic treatments, SFN’s ability to induce different types of cell death delineates it as a promising anticancer agent.

**Abstract:**

In recent years, natural compounds have emerged as inducers of non-canonical cell death. The isothiocyanate sulforaphane (SFN) is a well-known natural anticancer compound with remarkable pro-apoptotic activity. Its ability to promote non-apoptotic cell-death mechanisms remains poorly investigated. This work aimed to explore the capacity of SFN to induce non-apoptotic cell death modalities. SFN was tested on different acute myeloid leukemia cell lines. The mechanism of cell death was investigated using a multi-parametric approach including fluorescence microscopy, western blotting, and flow cytometry. SFN triggered different cell-death modalities in a dose-dependent manner. At 25 μM, SFN induced caspase-dependent apoptosis and at 50 μM ferroptosis was induced through depletion of glutathione (GSH), decreased GSH peroxidase 4 protein expression, and lipid peroxidation. In contrast, necroptosis was not involved in SFN-induced cell death, as demonstrated by the non-significant increase in phosphorylation of receptor-interacting protein kinase 3 and phosphorylation of the necroptotic effector mixed lineage kinase domain-like pseudokinase. Taken together, our results suggest that the antileukemic activity of SFN can be mediated via both ferroptotic and apoptotic cell death modalities.

## 1. Introduction

Worldwide cancer incidence continues to rise, causing a great deal of mental, social, and financial pressure on patients, families, societies, and health systems [1]. Although current anticancer therapies are increasingly efficient, tumor cells have developed the ability to evade apoptosis, leading to tumor growth and resistance against anticancer treatments [2]. Besides apoptosis, other non-apoptotic (or non-canonical) forms of programmed cell death (PCD) such as ferroptosis and necroptosis are increasingly well understood and show anticancer potential.

Non-canonical cell-death pathways differ from apoptosis from a morphological, biochemical, situational, and functional point of view and are generally caspase-independent [3]. Ferroptosis is an iron-dependent cell death pathway, associated with the blockage of the proliferation in multiple cancer cell models [4]. Ferroptosis is characterized by the intracellular accumulation of lipid peroxides caused by the direct inhibition of glutathione peroxidase 4 (GPX4). Alternatively, the accumulation of lipid peroxides can be due to the inhibition of the X_c_^_^ system or the depletion of intracellular glutathione (GSH) [5]. GPX4 is responsible for reducing hydrogen peroxides, organic hydroperoxides, or lipid peroxides into water or alcohols through the conversion of GSH into oxidized GSH (GSSG). Therefore, the inhibition of the GPX4 activity prompts lipid reactive oxygen species (ROS) accumulation, triggering ferroptotic cell death [5]. Necroptosis is regulated by receptor-interacting protein (RIP)1, RIP3, and mixed lineage kinase domain-like pseudokinase (MLKL) [6]. In an apoptosis-deficient environment, when caspase-8 is inhibited, RIP1 is phosphorylated and interacts with RIP3, promoting its phosphorylation. Phosphorylated RIP3 phosphorylates MLKL, inducing its oligomerization and translocation to the plasma membrane to execute necroptotic cell death [6,7].

Over 50% of anticancer drugs are derived from natural compounds or scaffolds found in nature [8]. Naturally derived compounds are helpful for preventive and therapeutic uses, since they may regulate many molecular targets involved in neoplastic transformation. In addition, several natural compounds trigger non-canonical cell-death pathways [9].

Most cruciferous vegetables are an important source of sulforaphane (SFN), an isothiocyanate displaying significant antitumor activity against various human neoplasms, validated through in vitro, in vivo, and clinical trials [10,11,12,13,14]. SFN can prevent, delay or reverse preneoplastic lesions, and inhibit tumor promotion and progression [13], and shows even a partial selectivity towards leukemia cancer cells compared to non-transformed lymphocytes [15,16].

SFN induces apoptosis, autophagy, and cell-cycle arrest, inhibits histone deacetylases (HDAC), and blocks angiogenesis and metastasis formation [13,14,17,18,19]. Specifically, in acute myeloid leukemia (AML) U-937 cells, SFN induced apoptosis by disturbing mitochondrial homeostasis and promoting the generation of ROS [20], and by regulating microRNA-155 levels [21]. Furthermore, some studies reported the ability of SFN to induce mitotic catastrophe [22,23,24], another type of non-canonical PCD. Interestingly, Iida and colleagues demonstrated that SFN triggers ferroptosis in NCI-H69 small-cell lung cancer cells [25]. This finding paves the way to the possibility that SFN can trigger apoptosis and multiple non-apoptotic cell deaths. Its pleiotropic nature prompted us to explore its ability to trigger non-canonical cell death in two AML cell lines with and without Fms-like tyrosine kinase (FLT)3 mutations.

## 2. Materials and Methods

### 2.1. Cell Cultures

U-937 (expressing wild-type FLT3) and MV4-11 [expressing FLT3 with an internal tandem duplication (ITD)] human AML cell lines were purchased from the Deutsche Sammlung für Mikroorganismen und Zellkulturen, Braunschweig (DSMZ; Braunschweig, Germany). U-937 and MV4-11 cell lines were authenticated using DNA profiling with the short tandem repeat method performed by DSMZ. U-937 and MV4-11 cells were cultured in Roswell Park Memorial Institute (RPMI) 1640 medium (Lonza, Verviers, Belgium) supplemented with 10% heat-inactivated fetal calf serum (FCS; Merck Millipore, Darmstadt, Germany) and penicillin/streptomycin solution 100 U/mL (Merck Millipore, Darmstadt, Germany). All cells were maintained at 37 °C and 5% CO_2_ in a humidified atmosphere.

### 2.2. Chemicals and Treatments

SFN, necrostatin-1, ferrostatin-1, and N-acetylcysteine were purchased from Merck Millipore (Darmstadt, Germany); N-Benzyloxycarbonyl-Val-Ala-Asp (O-Me) fluoromethyl ketone (Z-VAD-FMK) was obtained from Calbiochem, EMD Millipore Corp. (Billerica, MA, USA); necrosulfonamide was purchased from Tocris Bioscience (Bristol, UK); (1S,3R)-RSL3 (RSL3) and SM164 were obtained from Cayman Chemical (Ann Arbor, MI, USA); tumor necrosis factor α (TNF-α) was purchased from PeproTech GmbH (Hamburg, Germany).

Exponentially growing cells were used for all experiments. Cells were treated with increasing concentrations of SFN (0–50 μM) for 1, 3, 6, 9, 16, or 24 h, depending on the experimental conditions. To evaluate the induction of non-canonical cell death pathways, cells were pre-treated for 1 h with different chemical inhibitors and then treated with SFN for 24 h. The following inhibitors were used: the pan-caspase inhibitor Z-VAD-FMK (50 μM), the RIP1 inhibitor necrostatin-1 (50 μM), the MLKL inhibitor necrosulfonamide (1 μM), and the inhibitor of ROS generation and lipid peroxidation ferrostatin-1 (1 μM), to inhibit apoptosis, necroptosis, and ferroptosis, respectively. TNF-α (50 ng/mL) + SM164 (500 nM) + Z-VAD-FMK (50 μM) were used as positive control for necroptosis induction. RSL3 (0.2 μM) was used as a positive control for ferroptosis induction.

### 2.3. Analysis of Nuclear Morphology by Fluorescence Microscopy

Nuclear morphology was investigated by fluorescence microscopy using a cell^M imaging station (Olympus, Aartselaar, Belgium) on cells incubated for 15 min at 37 °C with the DNA-specific dye Hoechst 33342 (1 μg/mL; Merck Millipore, Darmstadt, Germany) and stained with 1 μg/mL propidium iodide (PI; Merck Millipore, Darmstadt, Germany). For each sample, at least 100 cells were counted. Quantification of the percentage of apoptotic cells was performed based on their fragmented nuclei and the condensed chromatin at the periphery of the nucleus: cells with distinct condensed nuclei, segregated nuclei, and apoptotic bodies were counted as apoptotic; PI-stained cells with a round morphology and homogeneously stained nuclei were termed PI-positive (necrotic); cells excluding PI, were categorized as viable cells.

### 2.4. Measurement of Reduced GSH

Ellman’s Reagent [5,5′-dithiobis-(2-nitrobenzoic acid) or DNTB] was used to measure the intracellular content of GSH. DTNB reacts with GSH, producing 2-nitro-5-thiobenzoic acid and glutathione disulfide, a yellow-colored product measured at 405 nM [26] using an infinite^®^ F200 PRO spectrophotometer (Tecan, Männedorf, Switzerland). After treatment, cells were harvested, washed in 1× phosphate-buffered saline (PBS), and lysed with CelLytic^TM^ reagent (Merck Millipore, Darmstadt, Germany). Protein concentration was measured using the Bradford assay with an infinite^®^ F200 PRO plate reader (Tecan, Männedorf, Switzerland) and extrapolated using the standard curve obtained from bovine serum albumin (BSA). An equal protein amount (minimum 20 μg) for each sample was used to measure GSH content. Cell lysates were deproteinized using trichloroacetic acid (TCA) and then centrifuged at 14,000 rounds per minute (RPM) for 5 min to remove the precipitated protein. 50 μL of supernatant was added to Tris-EDTA pH 8.9 and mixed with DTNB (0.01 M in methanol). GSH content was measured spectrophotometrically at 405 nm and expressed as fold change in treated cells compared to untreated cells. N-acetyl cysteine (NAC, 5 mM) was used as an internal control for the assay.

### 2.5. Flow Cytometry Analysis of GPX4 Protein Expression

After 24 h of treatment with SFN, 10^6^ cells of each condition were fixed with 4% formaldehyde and permeabilized using 90% cold methanol. Samples were then washed in wash buffer (WB; 1× PBS containing 1% BSA) and incubated for 1 h at 4 °C with the primary anti-GPX4 antibody (1:150; no. PA5-109274, Invitrogen, Thermo Fisher Scientific, Rockford, IL, USA). Next, cells were washed in WB and stained with the respective anti-rabbit secondary antibody (1:200; no. A21244, Invitrogen, Thermo Fisher Scientific, Rockford, IL, USA). After 1 h of incubation at 4 °C, cells were washed and then analyzed by flow cytometry, recording the mean fluorescence intensity (MFI) values. GPX4 protein expression was indicated as the fold change in treated cells compared to untreated cells.

### 2.6. Malondialdehyde Assay

Levels of malondialdehyde (MDA) were measured with the thiobarbituric acid reactive substances (TBARS) spectrophotometric assay using 1,1′,3,3′-tetraethoxypropane as a standard, as previously described [27]. Briefly, U-937 cells (1.5 × 10^6^) were resuspended in a 0.25 mL solution containing 1.15% KCl (*w*/*v*). The reaction mixture contained 0.25 mL of cell suspension, 0.1 mL of 8.1% sodium dodecyl sulfate (SDS), 1.5 mL of 20% acetic acid solution, and 1.5 mL of 0.8% aqueous solution of thiobarbituric acid. This mixture was diluted to 4 mL with distilled water and heated at 95 °C for 60 min. After cooling, 1.0 mL of distilled water and 5.0 mL of n-butanol were added, and the mixture was shaken vigorously. After centrifugation, the absorbance of the organic layer was measured at 532 nm.

### 2.7. Whole Cell Extracts and Immunoblotting

For the preparation of whole-cell extracts, cells were harvested, washed in ice-cold 1× PBS, and lysed in 1× Mammalian Protein Extraction Reagent (M-PER^®^; Thermo Fisher Scientific, Erembodegem-Aalst, Belgium) or radioimmunoprecipitation assay (RIPA) buffer (Merck Millipore, Darmstadt, Germany) supplemented with 1× protease inhibitor cocktail (Complete EDTA-free; Roche, Basel, Switzerland, or Merck Millipore, Darmstadt, Germany) and 1× phosphatase inhibitor (PhosSTOP^TM^; Roche, Basel, Switzerland) according to the manufacturer’s instructions. Protein concentration was measured using the Bradford assay (Bio-Rad, Hercules, CA, USA) or bicinchoninic acid (BCA) Protein Assay Kit (Thermo Fisher Scientific, Rockford, IL, USA) by a SpectraMax ID3 Multi-Mode Microplate Reader (Molecular Devices, LLC, San Jose, CA, USA) or VICTOR Multilabel plate reader (PerkinElmer, Wellesley, MA, USA) and extrapolated using the standard curve obtained from BSA. Proteins were aliquoted and stored at −80 °C. Proteins (20 or 30 μg) were separated by SDS-polyacrylamide gel electrophoresis (PAGE) and transferred onto a 0.2/0.45 nm pore size HybondTM-P membrane (GE Healthcare, Diegem, Belgium) or nitrocellulose membrane (Thermo Fisher Scientific, Rockford, IL, USA). Membranes were then incubated in 1× PBS or Tris-buffered saline (TBS) supplemented with 0.1% Tween (PBS-T, or TBS-T) containing the appropriated blocking agent (5% non-fat dry milk (NFDM) or 5% BSA) with the following primary antibodies: anti-caspase-3 (no. 9668, Cell Signaling, Danvers, MA, USA), anti-PARP-1 (no. 9542, Cell Signaling, Danvers, MA, USA; no. 53643, Santa Cruz Biotechnology, Dallas, TX, USA), anti-RIP3 (phospho S227, no. 93654, Cell Signaling, Danvers, MA, USA), anti-MLKL (phospho S358, no. 187091, Abcam, Cambridge, UK) and anti-β actin (no. A2228, Merck Millipore, Darmstadt, Germany; no. MA1-140, Thermo Fisher Scientific, Rockford, IL, USA). After washing with PBS-T or TBS-T, blots were incubated with anti-rabbit (no. 6721, Abcam, Cambridge, UK or no. NA9340, GE Healthcare, Diegem, Belgium) or anti-mouse (no. A28177, Thermo Fisher Scientific, Rockford, IL, USA) horseradish peroxidase-conjugated secondary antibody in PBS-T or TBS-T containing 5% NFDM or 5% BSA. After washing, proteins of interest were detected with Amersham^TM^ ECL^TM^ Prime Western Blotting Detection reagent (GE Healthcare, Roosendaal, The Netherlands) or SuperSignal™ West Pico PLUS Chemiluminescent Substrate (Thermo Fisher Scientific, Rockford, IL, USA) using the ImageQuant^TM^ LAS 4000 camera system (GE Healthcare, Roosendaal, The Netherlands) or ChemiDoc XRS + Gel Imaging System (Bio-Rad, Hercules, CA, USA).

### 2.8. Statistical Analysis

All experiments are expressed as the mean ± SEM of at least two or three independent experiments. Statistical analyses were assessed using one-way or two-way ANOVA tests, and Tukey, Dunnett, or Bonferroni were used as post-tests using GraphPad InStat 6.0 version (GraphPad Prism, San Diego, CA USA); *p*-values below 0.05 were considered significant and represented as * *p* < 0.05, ** *p* < 0.01, *** *p* < 0.001, and **** *p* < 0.0001.

## 3. Results and Discussion

### 3.1. SFN Induces Apoptosis in U-937 and MV4-11 Cells

**Figure 1:**
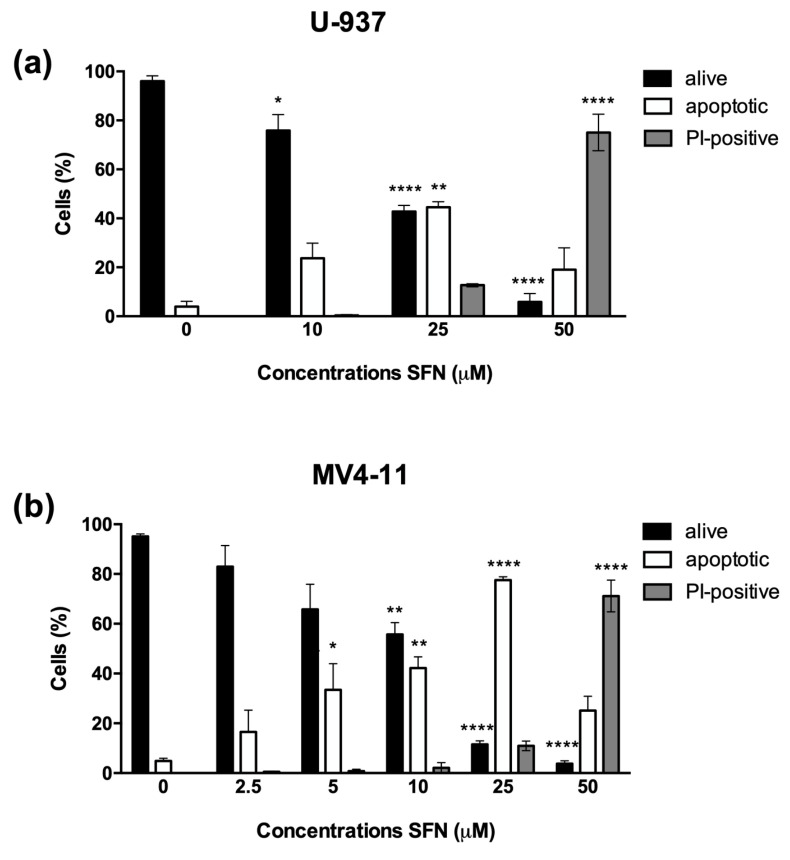
SFN treatment induces different cell death mechanisms in a dose-dependent manner. Evaluation of nuclear morphology of U-937 (**a**) and MV4-11 (**b**) cells after 24 h treatment with increasing concentrations of SFN. * *p* < 0.05; ** *p* < 0.01; **** *p* < 0.0001 versus untreated cells.

SFN dose-dependently decreased cell viability of U-937 and MV4-11 AML cells. To evaluate the mechanisms involved in the cytotoxic activity of SFN, we analyzed the nuclear morphology by fluorescence microscopy after Hoechst/PI staining of cells treated with increasing concentrations of SFN for 24 h. A dose-dependent increase in the apoptotic cell fraction was observed for both cell lines. The percentage of PI-positive cells never exceeded 10% of the total cell population for SFN concentrations up to 25 μM. At 50 μM, the fraction of PI-positive cells increased to 70%, suggesting the involvement of a different mechanism of cell death (Figure 1).

The pro-apoptotic activity of SFN was more marked on MV4-11 cells compared to U-937 cells. Based on current data, this different response to SFN on the two tested cell lines cannot be fully explained. However, what clearly distinguishes them is their different expression of FLT3 kinase: if U-937 cells express the wild-type form, MV4-11 cells express exclusively the mutated form with ITD [28]. FLT3-ITD mutation results in the constitutive activation of FLT3 kinase and of the downstream signaling cascades, such as the signal transducer and activator of transcription 5 (STAT5) [29]. FLT3-ITD positive cells have higher levels of active and phosphorylated STAT5 [30] and are more sensitive to STAT5 inhibition [29]. Given that SFN suppresses STAT5 activity on human chronic myeloid leukemia K562 cells [31], we may speculate that SFN activity on STAT5 could contribute to an increased cytotoxic response in FLT3-ITD positive MV4-11 cells. Of note, also other ITCs were more active on FLT3-ITD positive cells [32]. Alternatively, it may also be assumed that since one of the main SFN’s pro-apoptotic mechanisms is the generation of ROS [33], the higher levels of endogenous ROS expressed by cells carrying the FLT3-ITD mutation [30] could boost the induction of apoptosis by SFN. Additional studies are needed to corroborate both these hypotheses, but they fall beyond the aim of this work.

Many studies have reported the pro-apoptotic activity of SFN, mainly due to its ability to modulate several molecular targets involved in the apoptotic machinery [13,17,18,19]. Apoptosis has been observed in multiple cancer cell models after exposure to a wide range of SFN concentrations. Our observations suggest that the concentrations of SFN promoting apoptosis depend on the experimental model on which the ITC is tested and on the time of exposure. For example, SFN (10 and/or 20 μM) triggered apoptosis in osteosarcoma [34], bladder [35,36] and prostate cancer cells [37], and breast cancer cells succumbed to apoptosis after SFN 5–25 μM treatment [38], whereas in liver cancer, cell apoptosis has been observed after the exposure to SFN 20 and 40 μM [39]. In Jurkat lymphoblastic leukemia cells, SFN promoted apoptosis at the concentration of 30 μM [12], which is in line with our results obtained on U-937 and MV4-11 AML cells. However, in Jurkat cells, SFN 30 μM also increased the necrotic cell fraction, concomitantly with apoptosis [12]. Mouse sarcomatoid mammary carcinoma F3II cells exposed to SFN 15 μM showed the characteristic features of mitotic catastrophe, along with apoptosis [23].

**Figure 2:**
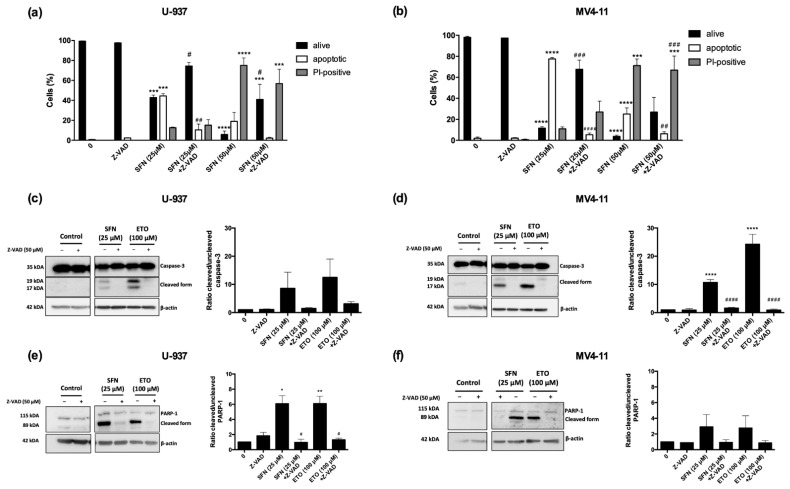
SFN at relatively low concentrations induces apoptotic cell death. Evaluation of nuclear morphology of U-937 (**a**) and MV4-11 (**b**) cells pre-treated for 1 h with Z-VAD-FMK (Z-VAD) and treated for 24 h with SFN; evaluation by Western Blotting of caspase-3 (**c**,**d**) and PARP-1 (**e**,**f**) activation on U-937 (**c**,**e**) and MV4-11 cells (**d**,**f**) pre-treated for 1 h with Z-VAD-FMK (Z-VAD) and treated for 24 h with SFN 25 μM: left panels show representative Western Blot results of caspase-3 and PARP-1 cleavage; right panels show the ratio of cleaved/uncleaved levels of caspase-3 and PARP-1. Cells treated with etoposide (ETO) 100 μM for 4 h (U-937) and 24 h (MV4-11) were used as positive control. * *p* < 0.05; ** *p* < 0.01; *** *p* < 0.001; **** *p* < 0.0001 versus untreated cells. # *p* < 0.05; ## *p* < 0.01; ### *p* < 0.001; #### *p* < 0.0001 versus Z-VAD-FMK untreated cells.

Then we analyzed the involvement of caspases in SFN-induced cell death. For this purpose, U-937 and MV4-11 cells were pre-incubated with the pan-caspase inhibitor Z-VAD-FMK and then treated with SFN (25 and 50 μM) for 24 h. In MV4-11 and U-937 cells, the pre-treatment with Z-VAD-FMK almost totally abrogated apoptosis induced by lower concentrations of SFN (25 μM): the percentage of apoptotic cells indeed decreased from about 40–70% to 5% (Figure 2a,b, Figures S1 and S2). Its pro-apoptotic activity was confirmed by caspase-3 and poly-(ADP-ribose)-polymerase-1 (PARP-1) cleavage in both cell lines after 24 h of treatment with SFN 25 μM (Figure 2c–f and Figures S3–S6). In contrast, the pre-treatment with Z-VAD-FMK did not affect the cell viability of U-937 and MV4-11 cells exposed to higher concentrations (50 μM) of SFN (Figure 2a,b, Figures S1 and S2), suggesting the involvement of other forms of cell death.

Numerous natural compounds were shown to activate multiple cell death pathways [40,41,42,43,44,45,46,47,48]. In some cases, the same compound induces different cell death pathways depending on the tested concentration. For example, in U-937, a low concentration of shikonin (1 μM) induces apoptosis, whereas 10 µM triggers necroptosis [44]. Columbianadin promotes apoptosis or necroptosis in HCT116 human colon cancer cells, at 25 μM and 50 μM, respectively [45]. Taxol, on its side, at low concentrations (35 nM) induces apoptosis in ASTC-a-1 human lung adenocarcinoma cells. Increasing the concentration up to 70 μM promotes a cell-death shift to paraptosis [46]. In other cases, different PCD pathways are activated at the same concentration. Arctigenin (5 μM; RPMI-2650 nasal septum carcinoma) [40], berberine (100 μM; OVCAR3 ovarian cancer) [48], neoalbaconol (40 mM; C-6661 nasopharyngeal carcinoma) [41] and tanshinone IIA (5/10 μg/mL; HepG2 hepatocellular carcinoma cells) [42] trigger both apoptosis and necroptosis. It is not surprising that these natural compounds elicit multiple modes of cell death, as they interact with cells in a pleiotropic and sometimes hormetic manner by modulating inflammation or antioxidant response [49,50].

### 3.2. Ferrostatin-1 and Necrostatin-1 Partially Prevent Cell Death and Switch the Type of Cell Death

Since high concentrations of SFN triggered caspase-independent cell death, we next investigated the potential involvement of non-apoptotic PCD pathways, particularly ferroptosis and necroptosis.

Firstly, we pre-treated U-937 and MV4-11 cells with the inhibitor of ROS generation and lipid peroxidation ferrostatin-1, the RIP1 inhibitor necrostatin-1, or the MLKL inhibitor necrosulfonamide with or without Z-VAD-FMK before the exposure to 50 μM SFN for 24 h.

**Figure 3:**
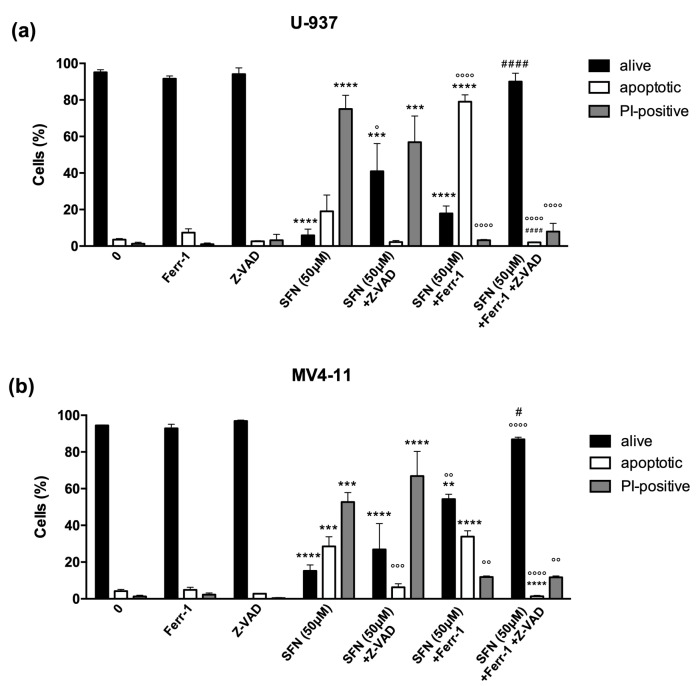
Ferrostatin-1 partially prevents cell death and switches the type of cell death induced by SFN 50 μM. Evaluation of nuclear morphology of U-937 (**a**) and MV4-11 (**b**) cells pre-treated for 1 h with ferrostatin-1 (ferr-1) with or without Z-VAD-FMK (Z-VAD) and then treated with SFN 50 μM for 24 h. ** *p* < 0.01; *** *p* < 0.001; **** *p* < 0.0001 versus untreated cells. ° *p* < 0.05; °° *p* < 0.01; °°° *p* < 0.001; °°°° *p* < 0.0001 versus cells treated with SFN. # *p* < 0.05; #### *p* < 0.0001 versus cells treated with SFN and ferr-1.

Pre-treatment with ferrostatin-1 induced a partial recovery of both U-937 and MV4-11 cell viability, with a more pronounced effect on MV4-11 (from 15.2% to 54.3%) than U-937 cells (from 5.9% to 17.8%). However, the most evident effect observed in both AML cell lines was that ferrostatin-1 switched the cell death mechanism from necrosis to apoptosis, as shown by the almost total conversion of SFN-induced necrotic cells into apoptotic ones. Indeed, pre-treatment with ferrostatin-1 reduced the fraction of necrotic cells from 75% to 3% and from 53% to 12% in U-937 and MV4-11 cells, respectively, accompanied by an increase in the apoptotic cell fraction from 19% to 79% and 28% to 34% in U-937 and MV4-11 cells, respectively (Figure 3, Figures S7 and S8). Accordingly, the pre-treatment of cells with ferrostatin-1 plus Z-VAD-FMK almost totally restored cell viability in both cell lines: the percentage of viable cells switched from 6% to 90% in U-937 cells and from 15% to 87% in MV4-11 cells (Figure 3, Figures S7 and S8). These results indicate that SFN may trigger ferroptosis at high concentrations. The partial recovery of U-937 viability by ferrostatin-1 may suggest that U-937 cells could still die by apoptosis after inhibition of ferroptosis.

**Figure 4:**
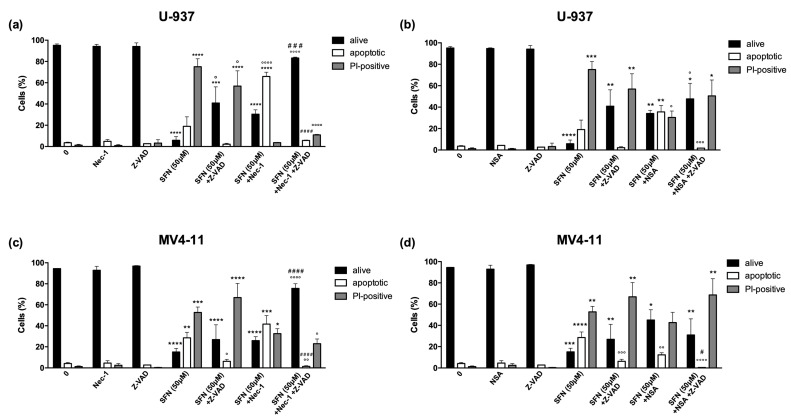
Necrostatin-1 and necrosulfonamide partially prevent cell death and switch the type of cell death induced by SFN 50 μM. Evaluation of nuclear morphology of U-937 (**a**,**b**) and MV4-11 (**c**,**d**) cells pre-treated for 1 h with necrostatin-1 (nec-1) (**a**,**c**) or necrosulfonamide (NSA) (**b**,**d**), with or without Z-VAD-FMK (Z-VAD), and then treated with SFN 50 μM for 24 h. * *p* < 0.05; ** *p* < 0.01; *** *p* < 0.001; **** *p* < 0.0001 versus untreated cells. ° *p* < 0.05; °° *p* < 0.01; °°° *p* < 0.001; °°°° *p* < 0.0001 versus cells treated with SFN. # *p* < 0.05; ### *p* < 0.001; #### *p* < 0.0001 versus cells treated with SFN and nec-1/NSA.

**Figure 5:**
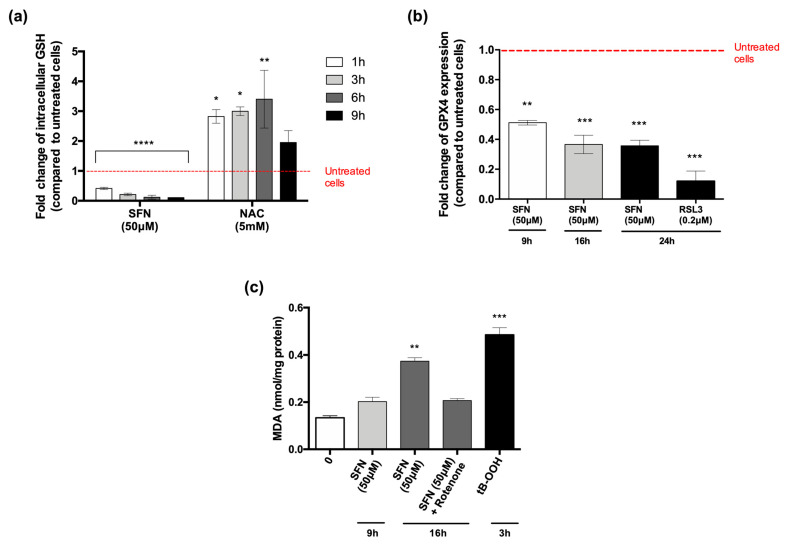
SFN 50 μM promotes ferroptosis in U-937 cells. Intracellular GSH content, expressed as fold change in SFN- or N-acetyl cysteine (NAC)-treated cells for 1–3–6–9 h compared to untreated cells. NAC 5 mM was used as internal control (**a**) Protein expression of GPX4, expressed as fold change in SFN-treated cells for 9–16–24 h compared to untreated cells. RSL3 0.2 μM was used as positive control (**b**) Levels of MDA (malondialdehyde) on U-937 cells treated with SFN 50 μM for 9 and 16 h with or without rotenone 0.1 μM. tB-OOH (tert-butyl hydroperoxide) 100 μM was used as positive control (**c**) * *p* < 0.05; ** *p* < 0.01; *** *p* < 0.001; **** *p* < 0.0001 versus untreated cells.

The pre-treatment of U-937 cells with necrostatin-1 led to a partial recovery of cell viability (from 5.9 to 30.4%) (Figure 4a and Figure S9). In comparison, the percentage of viable MV4-11 cells remained unchanged (from 15.3 to 25.9%) (Figure 4c and Figure S10). Furthermore, in this case, the conversion of necrotic cells into apoptotic ones was observed for both cell lines, and more markedly for U-937 cells. The fraction of PI-positive cells was reduced from 75.1% to 3.7% and from 52.7% to 32.5% in U-937 and MV4-11 cells, respectively; the fraction of apoptotic cells, however, increased from 19% to 65.9% and from 28.6% to 41.6% in U-937 and MV4-11 cells, respectively (Figure 4a, Figure 5c, Figures S9 and S10). Moreover, pre-treatment of both AML cell lines with necrostatin-1 and Z-VAD-FMK restored cell viability (from 5.9% to 83% in U-937 cells and from 15.2% to 75.6% in MV4-11 cells) (Figure 4a, Figure 5c, Figures S5 and S6), confirming the switch of PCD pathway.

Similar results were obtained by pre-treating cells with necrosulfonamide. As for necrostatin-1 treatment, necrosulfonamide induced only a partial recovery of cell viability: the percentage of viable cells increased from 5.9% to 34.1% in U-937 cells and from 15.2 to 45% in MV4-11 cells (Figure 4b,d). Instead, the conversion of necrotic cells into apoptotic ones was lower than that of necrostatin-1 pre-treatment on both cell lines (Figure 4b,d). In addition, in contrast to the other inhibitors, pre-treatment with necrosulfonamide and Z-VAD-FMK only partially restored U-937 and MV4-11 cell viability and did not have any effect on the fraction of necrotic cells, which was comparable to that observed in U-937 and MV4-11 cells exposed to SFN with or without the pre-treatment with Z-VAD-FMK (Figure 4b,d).

### 3.3. SFN Promotes Ferroptosis but Not Necroptosis in U-937 Cells

To investigate the molecular mechanisms underlying SFN-induced ferroptosis and necroptosis, we used U-937 cells, where both pathways appeared to be more markedly modulated by SFN.

GPX4 inhibits the formation of lipid peroxides and is one of the main enzymatic actors involved in the ferroptotic process [5]. Indeed, ferroptosis occurs after accumulation of lipid ROS. This event could be triggered by either GSH depletion, as GSH is an essential GPX4 cofactor, or GPX4 inhibition [5].

First, we evaluated the main processes involved in ferroptosis. As mentioned before, depletion of GSH could favor ferroptosis [51]. U-937 cells were exposed to SFN, and the intracellular levels of GSH were analyzed after 1–3–6–9 h of treatment. SFN caused a drastic and significant drop in GSH in a time-dependent manner: the intracellular levels of GSH were reduced by 60% at 1 h and 80% at 3 h, and, starting from 6 h of treatment, intracellular GSH was almost entirely depleted by SFN (Figure 5a).

We next analyzed GPX4 protein expression by flow cytometry. After 9, 16, and 24 h of treatment, SFN significantly reduced GPX4 protein expression. After 9 h, GPX4 protein expression was decreased by 51%, and even reduced by 63% and 64% after 16 h and 24 h, respectively (Figure 5b). However, whether the depletion of GSH and GPX4 modulation are related or constitute two separate mechanisms of ferroptosis induction is still undefined.

As a secondary, lipid peroxidation product, MDA is a stable marker used to assess the accumulation of lipid peroxides [52]. Therefore, we evaluated the intracellular levels of MDA in SFN-treated U-937 cells. SFN promoted lipid peroxidation in U-937 cells in a time-dependent manner. Indeed, the intracellular levels of MDA increased after 9 h (1.51-fold) and even more after 16 h (2.81-fold) in cells exposed to SFN, compared to untreated cells. Quantitatively, the amount of MDA was 0.20 and 0.37 nmol/mg protein after 9 and 16 h, respectively, compared to 0.13 nmol/mg protein of MDA in untreated cells (Figure 5c).

Additionally, to understand the role of mitochondria in SFN-induced ferroptosis, we tested whether the electron transfer chain (ETC) complex I inhibitor rotenone could inhibit the accumulation of lipid ROS induced by SFN. Co-treatment with SFN and rotenone for 16 h partially reduced but did not suppress SFN-induced lipid peroxidation (Figure 5c). The role of mitochondria in ferroptosis remains controversial. Gao et al. found that mitochondria-deficient cells were resistant to ferroptotic cell death induced by erastin or cysteine deprivation. Conversely, non-functioning mitochondria did not preclude RSL3-induced ferroptosis [53]. In a different study, Gaschler and colleagues showed that a panel of ferroptosis inducers, including erastin and RSL3, killed HT-1080 fibrosarcoma cells, regardless of the status of mitochondria (active or depleted), suggesting that mitochondria are dispensable for ferroptosis [54]. However, it should be considered that these apparent discrepancies may simply reflect the differences in the intoxication conditions rather than a contradictory role of mitochondria in this process. Mitochondria appear to be only partially involved in mediating the SFN-induced lipid oxidative stress in our experimental setting. Further studies are needed to understand whether this non-exhaustive inhibition of peroxidation is sufficient to block ferroptosis, and thus whether the mitochondrial activity is a critical event in ferroptosis induction by SFN.

**Figure 6:**
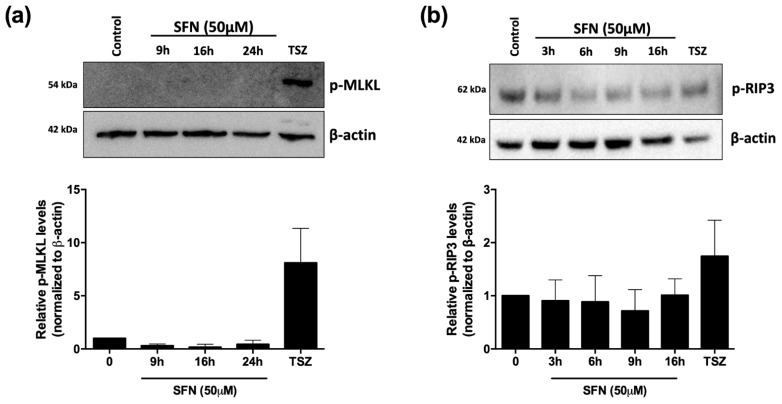
Necroptotic cell death is not activated by SFN 50 μM. Evaluation by Western Blot of phosphorylated protein levels for MLKL (p-MLKL; **a**) and RIP3 (p-RIP3; **b**) in U-937 cells treated with SFN 50 μM for the indicated time. U-937 cells treated with 50 ng/mL TNF-α + 500 nM SM-164 (S) + 50 µM Z-VAD-FMK (TSZ) for 4 or 6 h were used as positive control for RIP3 induction and MLKL phosphorylation. Upper panel shows representative Western Blot results of p-MLKL (**a**) and p-RIP3 (**b**); lower panel shows protein quantification expressed as relative levels of p-MLKL (**a**) and p-RIP3 (**b**) normalized to β-actin.

To investigate the involvement of necroptotic cell death in the cytotoxicity of SFN, we analyzed the protein expression of the key regulators of necroptosis. Firstly, we investigated the level of phosphorylated MLKL (p-MLKL), as phosphorylation of MLKL is crucial for the execution of the necroptotic process [55]. Results obtained indicate that at all tested time treatments (i.e., 9, 16, and 24 h), phosphorylation of MLKL takes place only in U-937 cells treated with a combination of TNF-α/SM-164/Z-VAD-FMK, the gold standard of necroptosis induction [55] (Figure 6a and Figure S11). To further confirm that necroptosis was not the type of cell death induced by SFN, we analyzed the expression of phosphorylated RIP3 (p-RIP3). SFN induced a non-significant increase in p-RIP3 protein expression (Figure 6b and Figure S12). Overall, although necrostatin-1 led to a partial recovery of cell viability, it seems that SFN cannot fully activate the necroptotic process, or that the activation is so mild that it cannot be completed. This could also explain why, by pre-treating cells with necrosulfonamide, we did not observe the same effect as the pre-treatment with necrostatin-1. In conclusion, we found that necroptosis is not involved in cell death induced by SFN at high concentrations.

As mentioned before, the potential involvement of additional non-apoptotic PCD pathways in the anticancer activity of SFN is poorly defined. Jackson et al. recorded that SFN (15 µM, 24 h) induced mitotic catastrophe in breast MCF-7 cancer cells, as observed by aberrant mitosis and micronucleation [22]. In mouse sarcomatoid mammary carcinoma F3II cells, SFN (15 µM, 36 h) blocked cell proliferation in the G2/M phase and promoted mitotic arrest with a premature cdc2 kinase activity [23]. In addition, suppression of endothelial cell proliferation by SFN (15 µM, 24 h) was related to the induction of mitotic catastrophe in bovine aortic endothelial cells. The authors demonstrated extensive micronucleation, aberrant cell-cycle progression with mitotic figures lacking complete and symmetrically bipolar mitotic spindle arrays, micronuclei, and diffuse cytoplasmic microtubule staining in SFN-treated cells [24].

A very recent study demonstrated that SFN (20 μM, 96 h) triggered ferroptosis in NCI-H69 small-cell lung cancer cells through the inhibition of mRNA and protein expression of the cystine/glutamate antiporter system X_c_^_^ (SLC7A11), leading to a reduction in intracellular GSH and increased lipid peroxidation [25]. As observed in NCI-H69 small-cell lung cancer cells, we found that SFN-induced ferroptosis was accompanied by a decrease in the intracellular GSH content and an increase in lipid peroxidation. In addition, we observed that SFN-induced ferroptosis is also linked to the downregulation of GPX4 protein expression. Four classes of ferroptosis inducers (FINs) have been described so far. Class I includes erastin and inactivates GPX4 through the direct or indirect depletion of its crucial cofactor GSH. RSL3 and all class II and III FINs directly inactivate GPX4, while class IV affects iron homeostasis [56]. This means that SFN could be a ferroptosis inducer belonging to both class I, possibly inhibiting system X_c_^_^ activity or directly depleting GSH levels, and class II, thanks to its inhibitory activity on GPX4.

Taken together, our results indicate that ferroptosis is involved in SFN-induced cell death. Moreover, ferrostatin-1 did not cause a total recovery of U-937-treated cells, but induced an almost complete conversion of SFN-induced necrosis into apoptosis. This evidence may indicate that, at higher SFN concentrations, ferroptosis is the predominant cell death pathway, while apoptosis only occurs when the ferroptotic process is inhibited. Accordingly, the inhibition of both ferroptosis and apoptosis almost totally rescued the cell viability of SFN-treated cells without necrosis.

## 4. Conclusions

Overall, our results indicate that SFN triggers different types of PCD in a concentration-dependent manner on the two tested AML cell lines. Deepening the molecular mechanisms on U-937 cells, we discovered that at lower concentrations, SFN induces apoptosis; at higher concentrations, SFN elicits ferroptosis, characterized by the depletion of intracellular GSH, the downregulation of GPX4 protein expression, and lipid peroxidation. Moreover, in addition to the concentration-dependent effect, SFN converted one cell death modality to another. Indeed, when ferroptosis is impaired, apoptosis is activated, minimizing the probability of resistance.

Ferroptosis represents a new efficient antitumor intervention strategy alternative to apoptosis. Of note, ferroptosis is an immunogenic type of cell death [57]. This means that cells dying by ferroptosis can stimulate the adaptive immune response and induce an effect comparable to an anticancer vaccine. Thus, SFN’s ability to induce ferroptosis and convert one PCD mechanism to another makes its use as an anticancer agent very appealing and paves the way for future clinical studies.

## Data Availability

The data presented in this study are available on request from the corresponding author.

## Supplementary Materials

The following are available online at https://www.mdpi.com/article/10.3390/cancers14010076/s1, Figure S1. Representative images of the analysis of nuclear morphology by fluorescent microscopy of U-937 cells, pre-treated or not with Z-VAD-FMK (Z-VAD), and then treated with 25 or 50 μM SFN for 24 h. Figure S2. Representative images of the analysis of nuclear morphology by fluorescent microscopy of MV4-11 cells, pre-treated or not with Z-VAD-FMK (Z-VAD), and then treated with SFN 25 or 50 μM for 24 h. Figure S3. Uncropped images (whole Western Blot images) showing bands overlaid with the molecular weight marker of the three replicates related to the protein expression of full and cleaved caspase-3, and respective protein expression of β-actin on U-937 cells. The ratio of cleaved/uncleaved caspase-3 is shown for three replicates. Figure S4. Uncropped images (whole Western Blot images) showing bands overlaid with the molecular weight marker of the two replicates related to the protein expression of full and cleaved PARP-1, and respective protein expression of β-actin of U-937. The ratio of cleaved/uncleaved PARP-1 is shown for the two replicates. Figure S5. Uncropped images (whole Western Blot images) showing bands overlaid with the molecular weight marker of the three replicates related to the protein expression of full and cleaved caspase-3, and respective protein expression of β-actin on MV4-11 cells. The ratio of cleaved/uncleaved caspase-3 is shown for three replicates. Figure S6. Uncropped images (whole Western Blot images) showing bands overlaid with the molecular weight marker of the two replicates related to the protein expression of full and cleaved PARP-1, and respective protein expression of β-actin on MV4-11 cells. The ratio of cleaved/uncleaved PARP-1 is shown for the two replicates. Figure S7. Representative images of the analysis of nuclear morphology by fluorescent microscopy of U-937 cells pre-treated with ferrostatin (Ferr-1) with or without Z-VAD-FMK (Z- VAD) and then treated with 50 μM SFN for 24 h. Figure S8. Representative images of the analysis of nuclear morphology by fluorescent microscopy of MV4-11 cells pre-treated with ferrostatin-1 (Ferr-1) with or without Z-VAD-FMK (Z-VAD) and then treated with 50 μM SFN for 24 h. Figure S9. Representative images of the analysis of nuclear morphology by fluorescent microscopy of U-937 cells pre-treated with necrostatin-1 (nec-1) with or without Z-VAD-FMK (Z-VAD) and then treated with 50 μM SFN for 24 h. Figure S10. Representative images of the analysis of nuclear morphology by fluorescent microscopy of MV4-11 cells pre-treated with necrostatin-1 (nec-1) with or without Z-VAD-FMK (Z-VAD) and then treated with SFN 50 μM 24 h. Figure S11. Uncropped images (whole Western Blot images) showing bands overlaid with the molecular weight marker of the three replicates related to phosphorylated MLKL (p-MLKL) protein levels and the respective protein expression of β-actin. Quantification is expressed as fold change versus untreated cells (normalized to β-actin). Figure S12. Uncropped images (whole Western Blot images) showing bands overlaid with the molecular weight marker of the three replicates related to phosphorylated RIP-3 (p-RIP3) protein levels compared to protein expression of β-actin. Quantification is expressed as fold change versus untreated cells (normalized to β-actin).
