# Supplementary material for: Discovery of Sulforaphane as an Inducer of Ferroptosis in U-937 Leukemia Cells: Expanding Its Anticancer Potential
Source: Cancers (Basel). 2021 Dec 24;14(1):76. doi: 10.3390/cancers14010076 (PMC8750507; doi:10.3390/cancers14010076)
Supplement: Supplementary file 1 [file cancers-14-00076-s001.zip › cancers-1480790-supplementary.pdf]

Supplementary Materials

Discovery of sulforaphane as an inducer of ferroptosis in U-937 leukemia cells: expanding its anticancer potential

Giulia Greco[1], Michael Schnekenburger[2], Elena Catanzaro[1], Eleonora Turrini[1], Fabio Ferrini[3], Piero Sestili[3], Marc Diederich[4], Carmela Fimognari[1*]

[1] Dipartimento di Scienze per la Qualità della Vita, Alma Mater Studiorum-Università di Bologna, Corso d'Augusto 237, 47921 Rimini, Italy
[2] Laboratoire de Biologie Moléculaire et Cellulaire du Cancer, Hôpital Kirchberg, 9 rue Edward Steichen, L-2540 Luxembourg, Luxembourg
[3] Dipartimento di Scienze Biomolecolari, Università degli Studi di Urbino Carlo Bo, 61029 Urbino, Italy
[4] Department of Pharmacy, College of Pharmacy, Seoul National University, 1 Gwanak-ro, Gwanak-gu, Seoul 08826, Korea
* Correspondence: carmela.fimognari@unibo.it

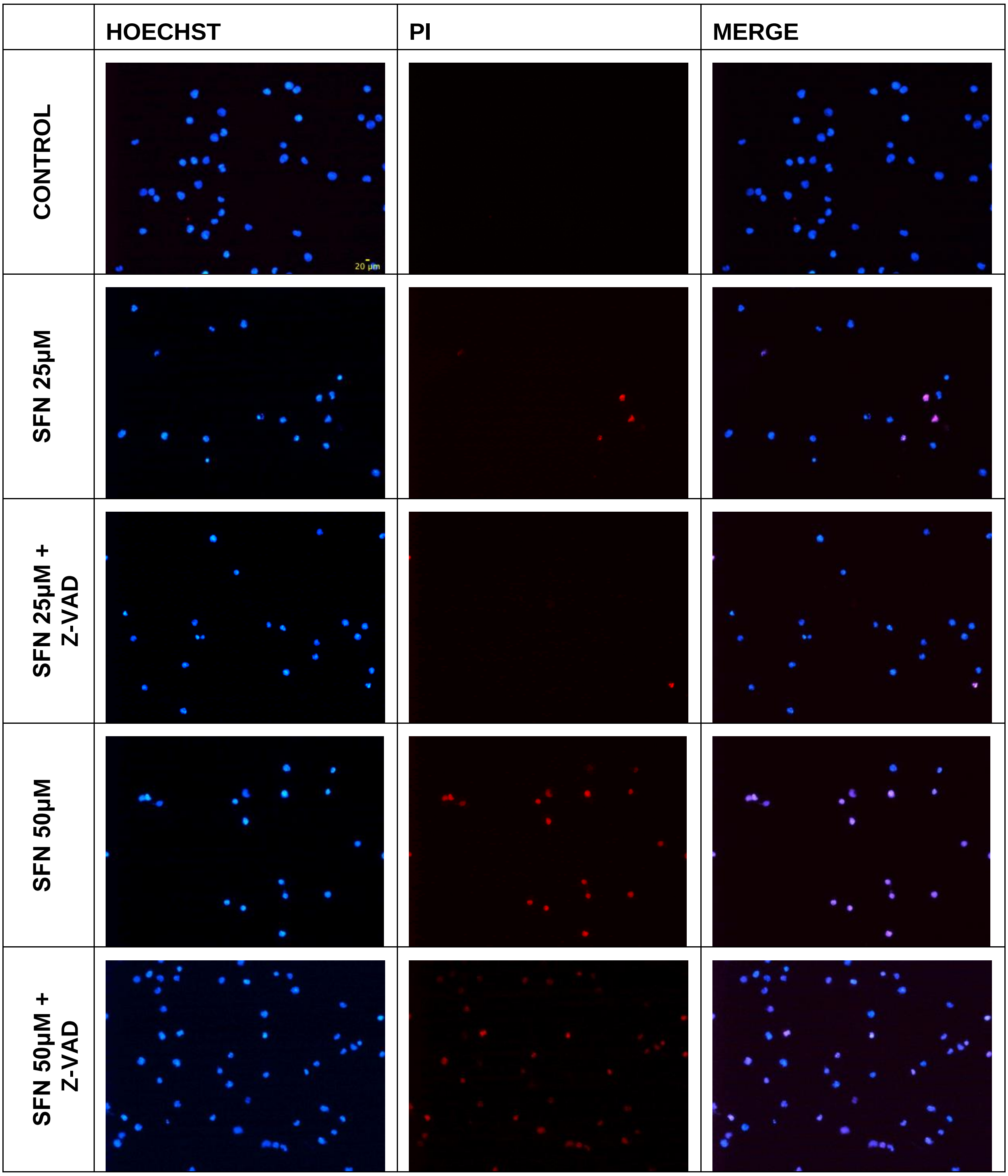

Figure S1. Representative images of the analysis of nuclear morphology by fluorescent microscopy of U-937 cells pre-treated or not with Z-VAD-FMK (Z-VAD) and then treated with SFN 25 or 50 µM for 24h.

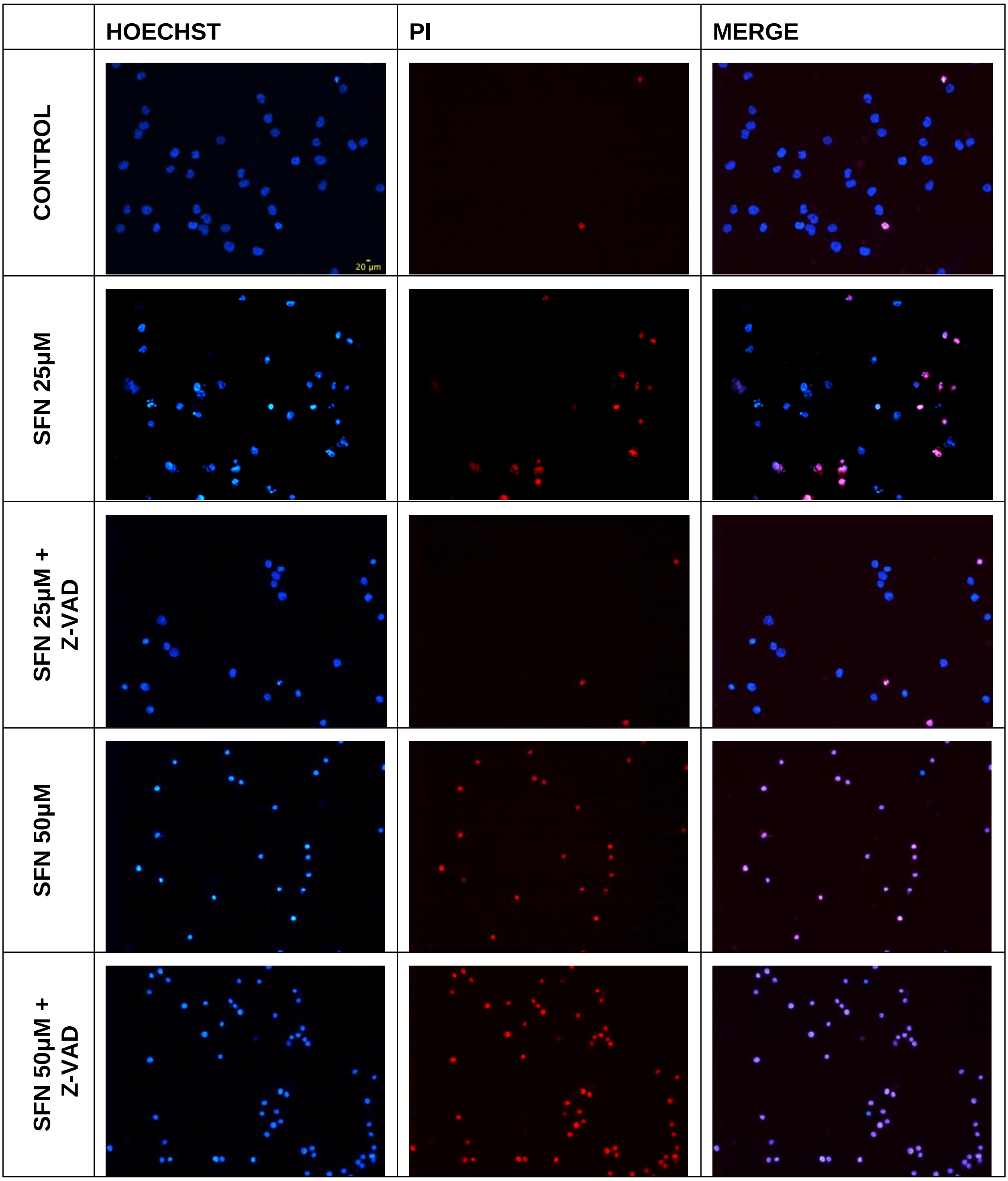

Figure S2. Representative images of the analysis of nuclear morphology by fluorescent microscopy of MV4-11 cells pre-treated or not with Z-VAD-FMK (Z-VAD) and then treated with SFN 25 or 50 μM for 24h.

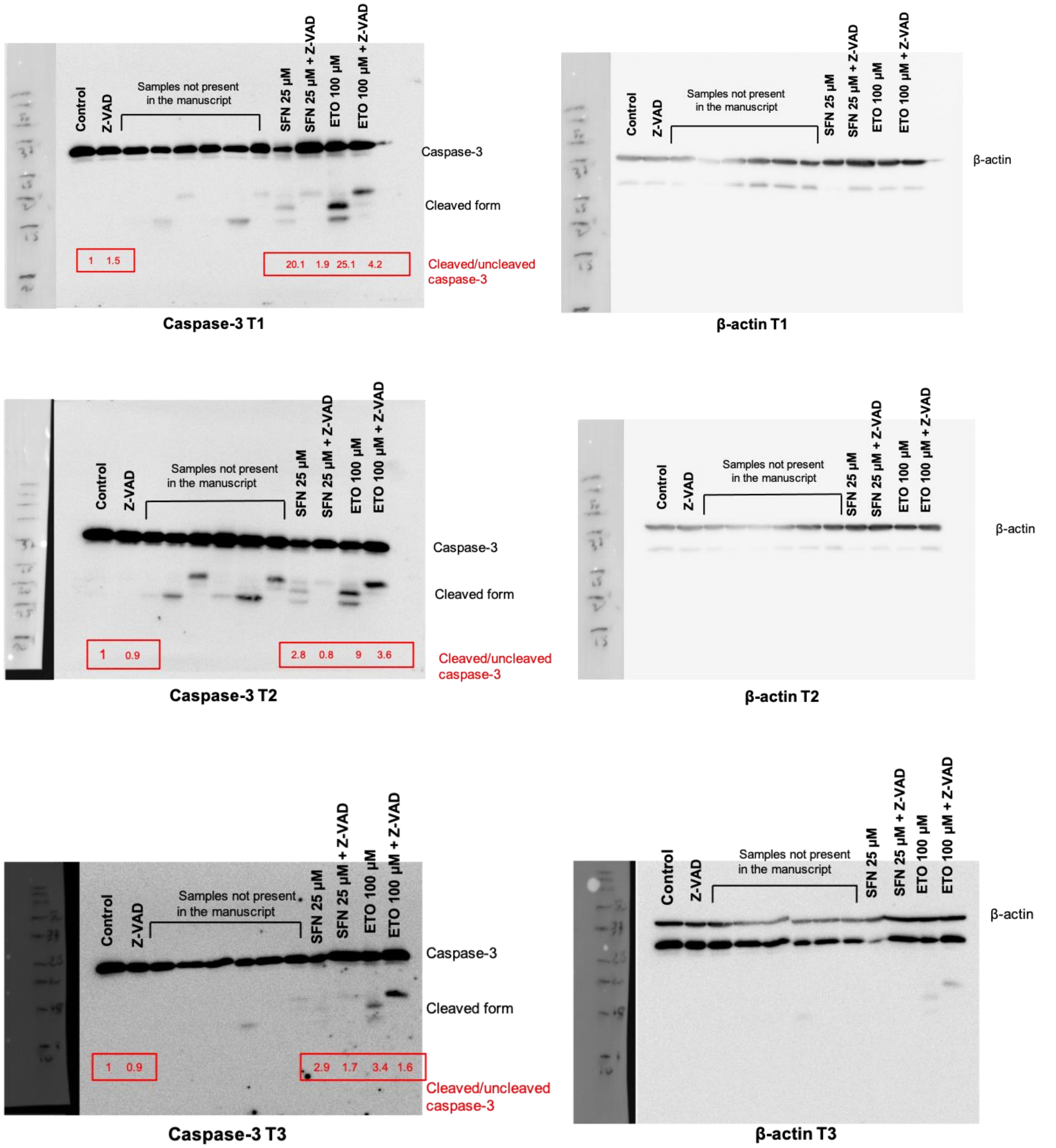

Figure S3. Uncropped images (whole Western Blot images) showing bands overlaid with the molecular weight marker of the three replicates related to the protein expression of full and cleaved caspase-3, and respective protein expression of β-actin on U-937 cells. The ratio of cleaved/uncleaved caspase-3 is showed for the three replicates.

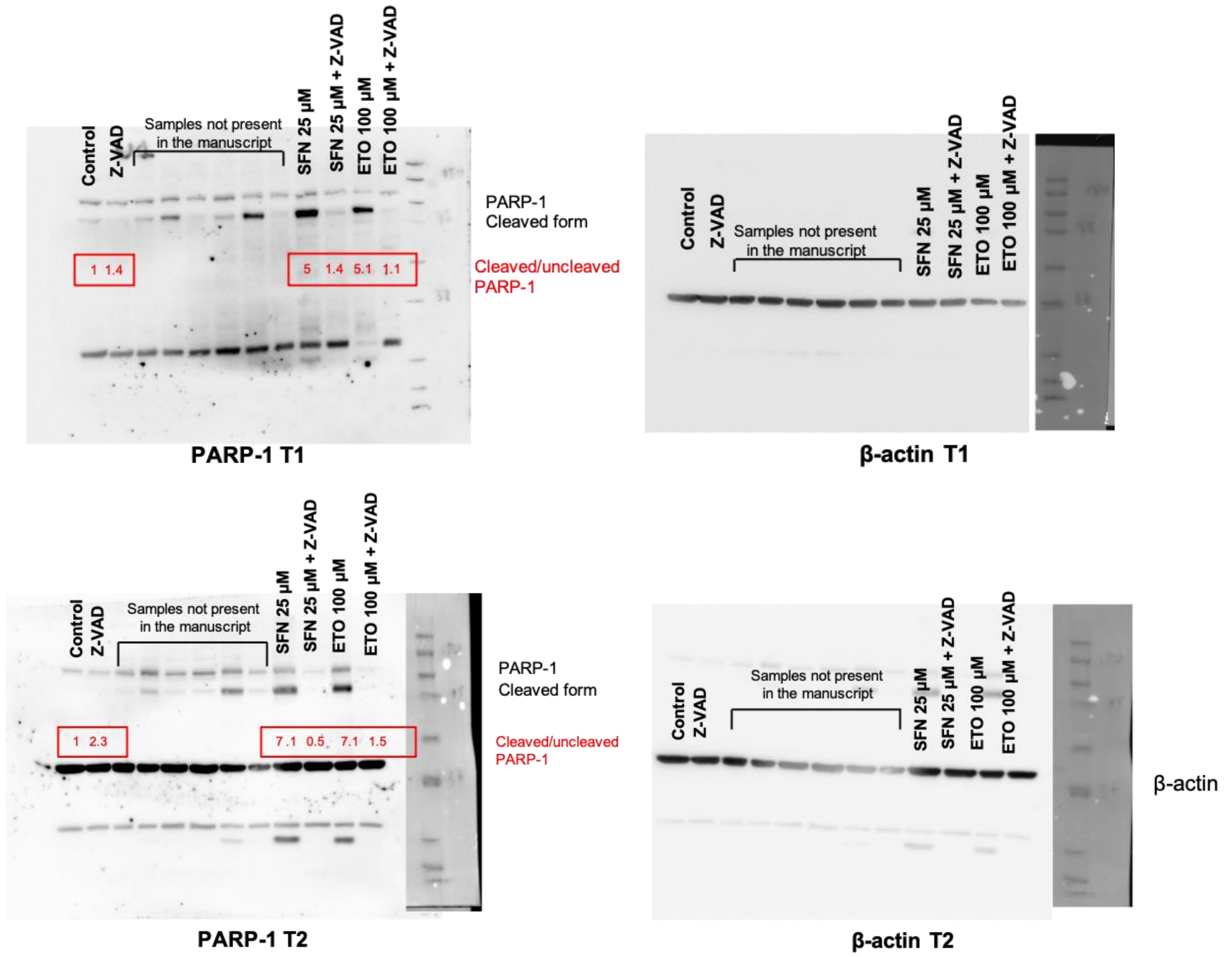

Figure S4. Uncropped images (whole Western Blot images) showing bands overlaid with the molecular weight marker of the two replicates related to the protein expression of full and cleaved PARP-1, and respective protein expression of β-actin of U-937 cells. The ratio of cleaved/uncleaved PARP-1 is showed for the two replicates.

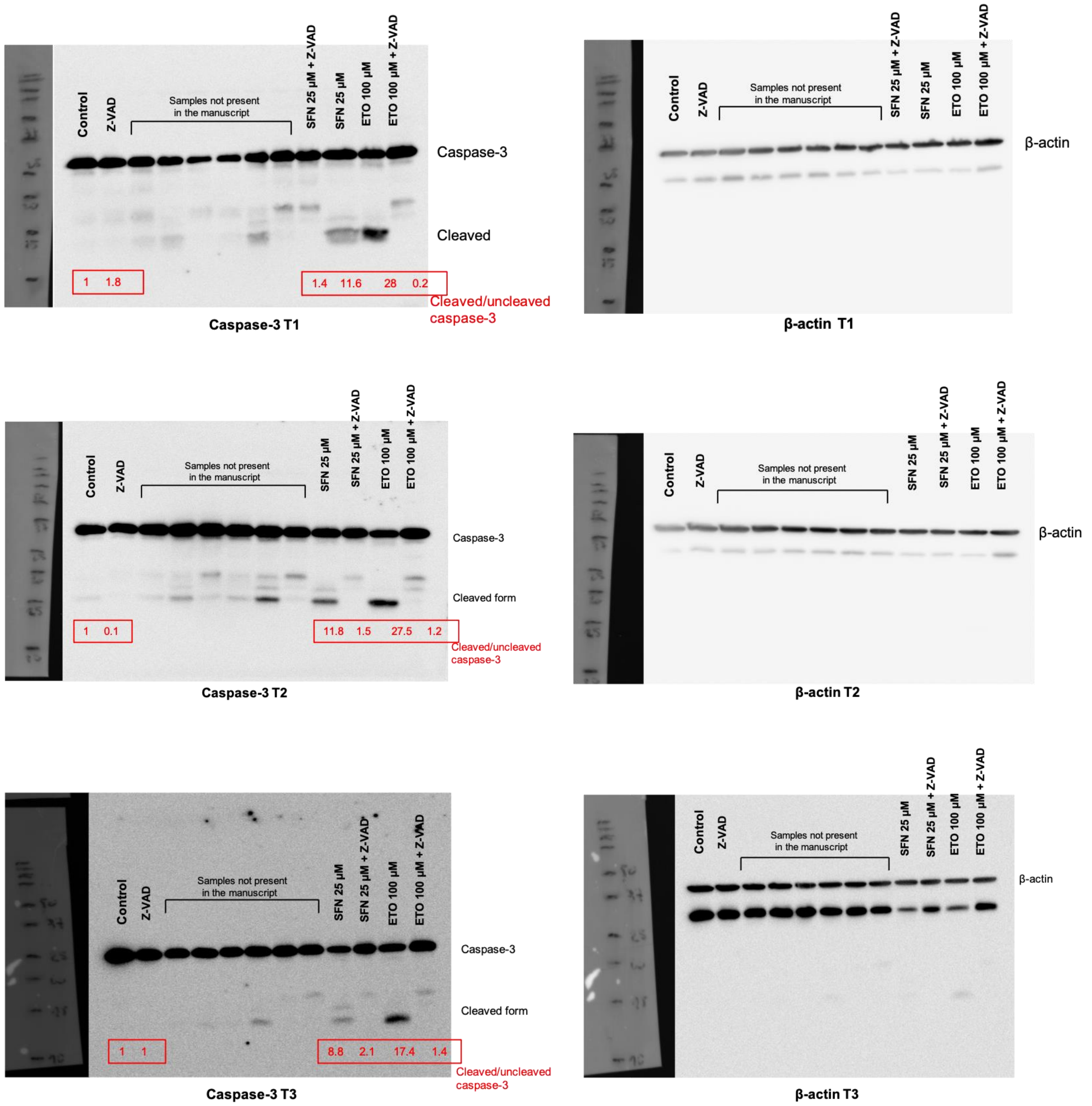

Figure S5. Uncropped images (whole Western Blot images) showing bands overlaid with the molecular weight marker of the three replicates related to the protein expression of full and cleaved caspase-3, and respective protein expression of β-actin on MV4-11 cells. The ratio of cleaved/uncleaved caspase-3 is showed for three replicates.

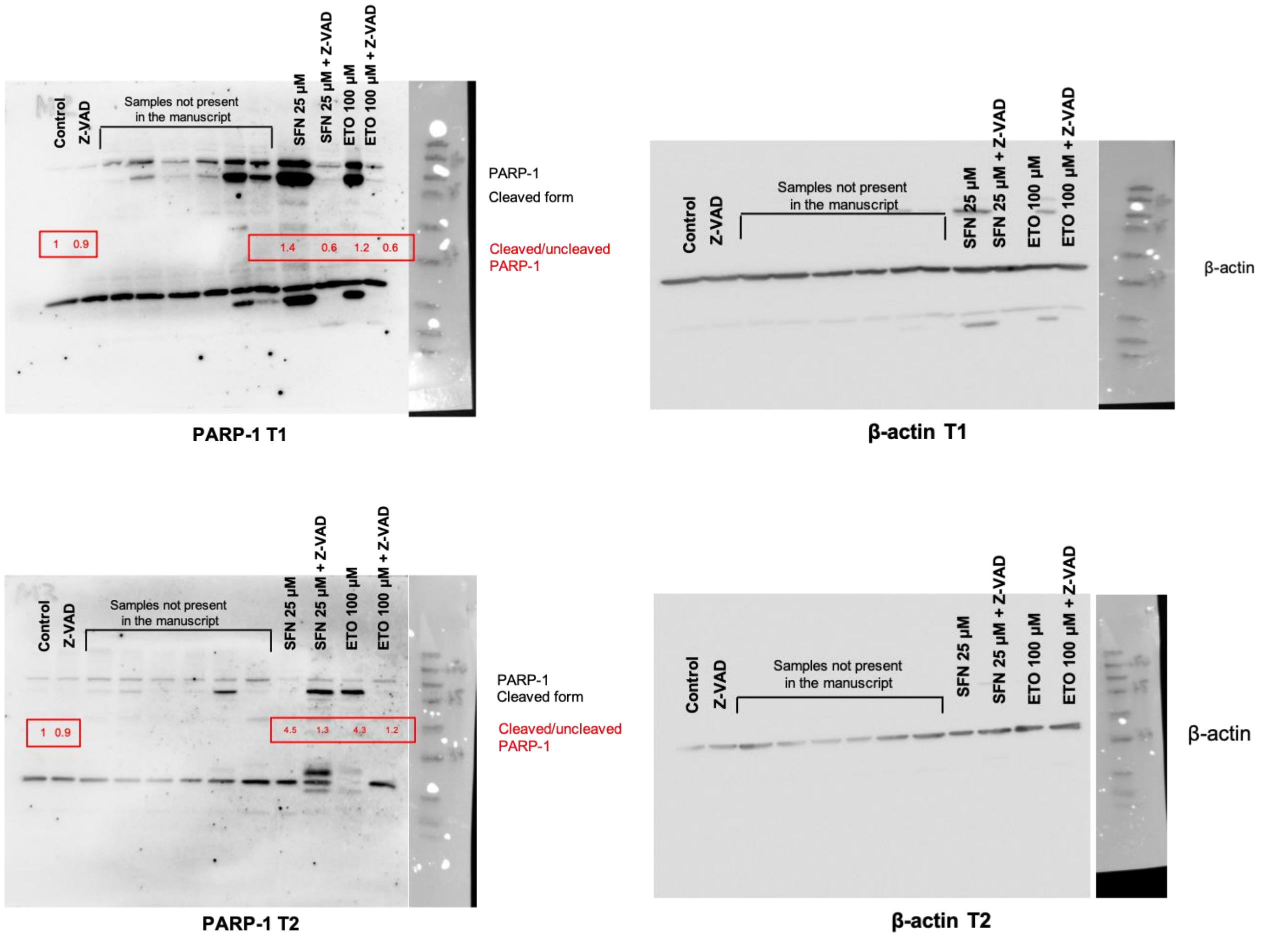

Figure S6. Uncropped images (whole Western Blot images) showing bands overlaid with the molecular weight marker of the two replicates related to the protein expression of full and cleaved PARP-1, and respective protein expression of β-actin on MV4-11 cells. The ratio of cleaved/uncleaved PARP-1 is showed for the two replicates.

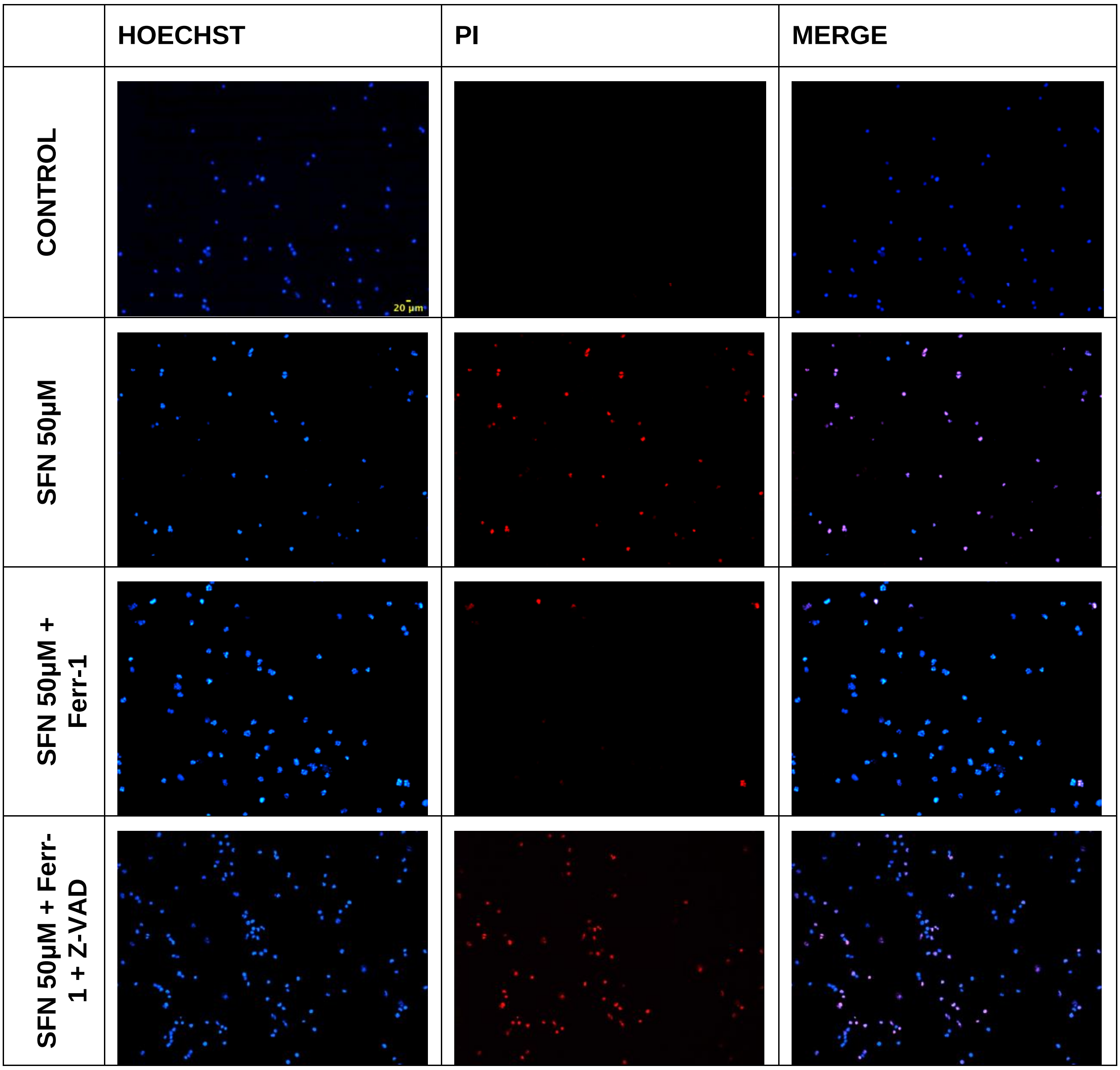

Figure S7. Representative images of the analysis of nuclear morphology by fluorescent microscopy of U-937 cells pre-treated with ferrostatin (Ferr-1) with or without Z-VAD-FMK (Z-VAD) and then treated with SFN 50 µM for 24h.

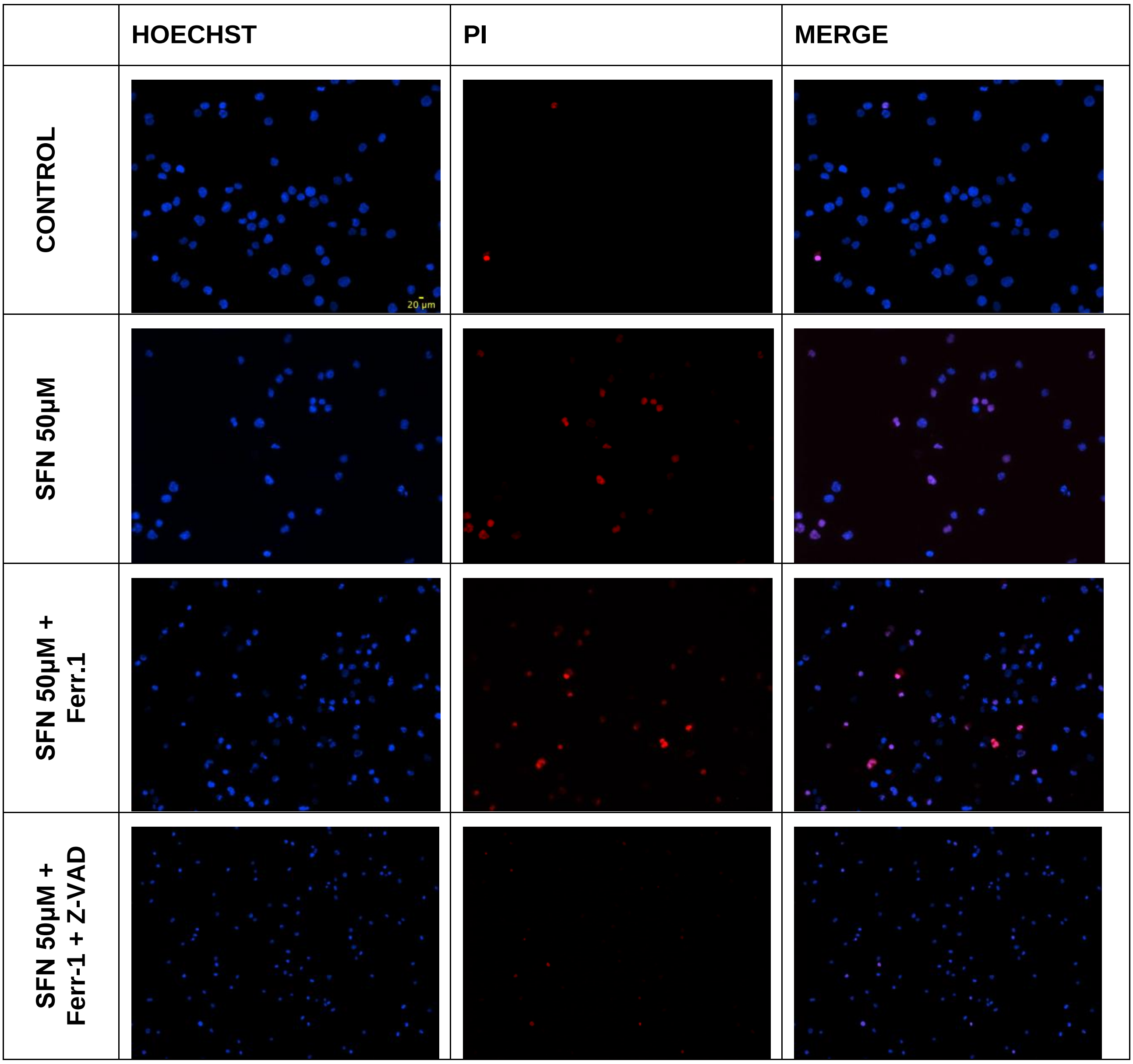

Figure S8. Representative images of the analysis of nuclear morphology by fluorescent microscopy of MV4-11 cells pre-treated with ferrostatin-1 (Ferr-1) with or without Z-VAD-FMK (Z-VAD) and then treated with SFN 50 µM for 24h.

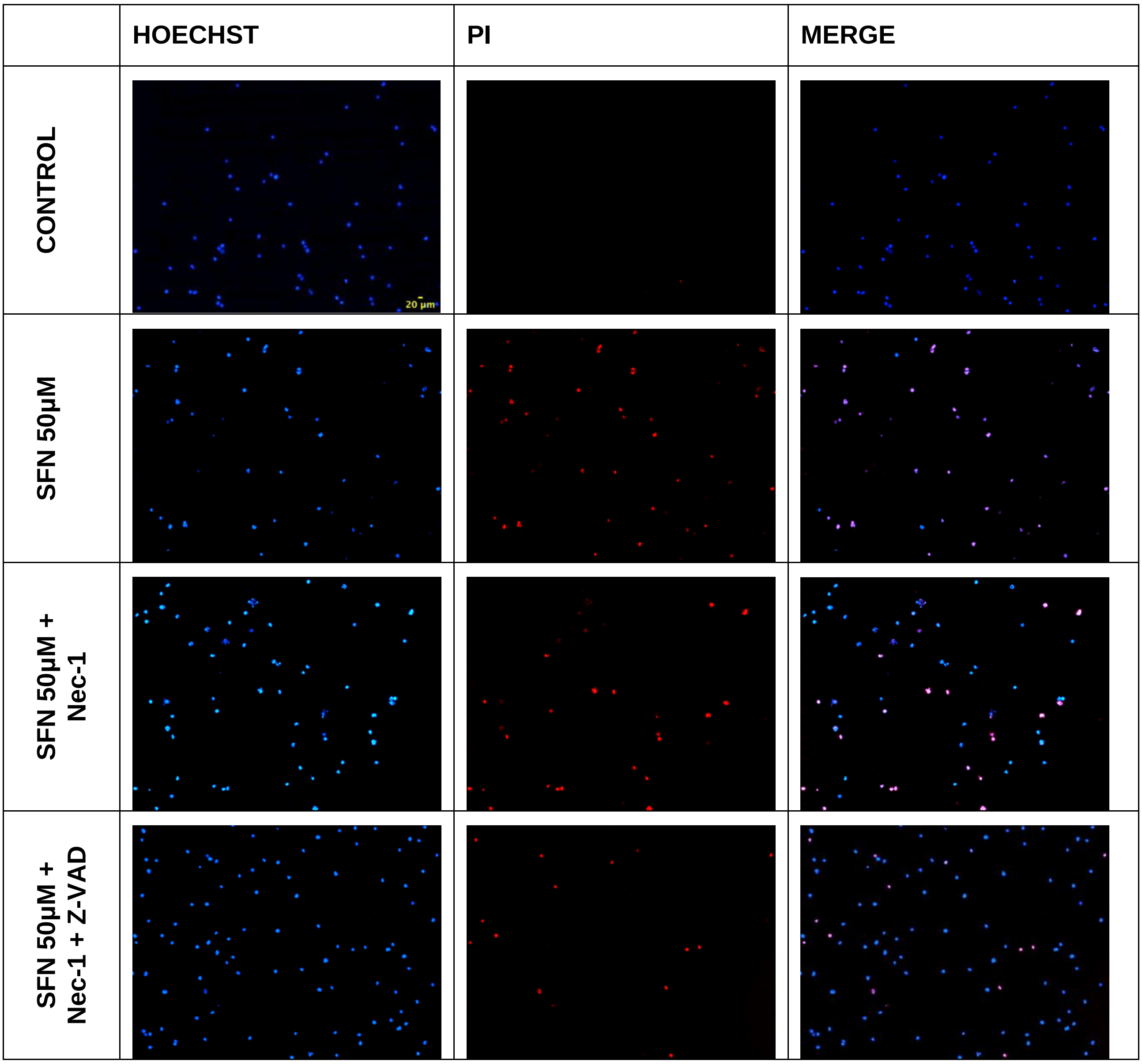

Figure S9. Representative images of the analysis of nuclear morphology by fluorescent microscopy of U-937 cells pre-treated with necrostatin-1 (Nec-1) with or without Z-VAD-FMK (Z-VAD) and then treated with SFN 50 μM for 24h.

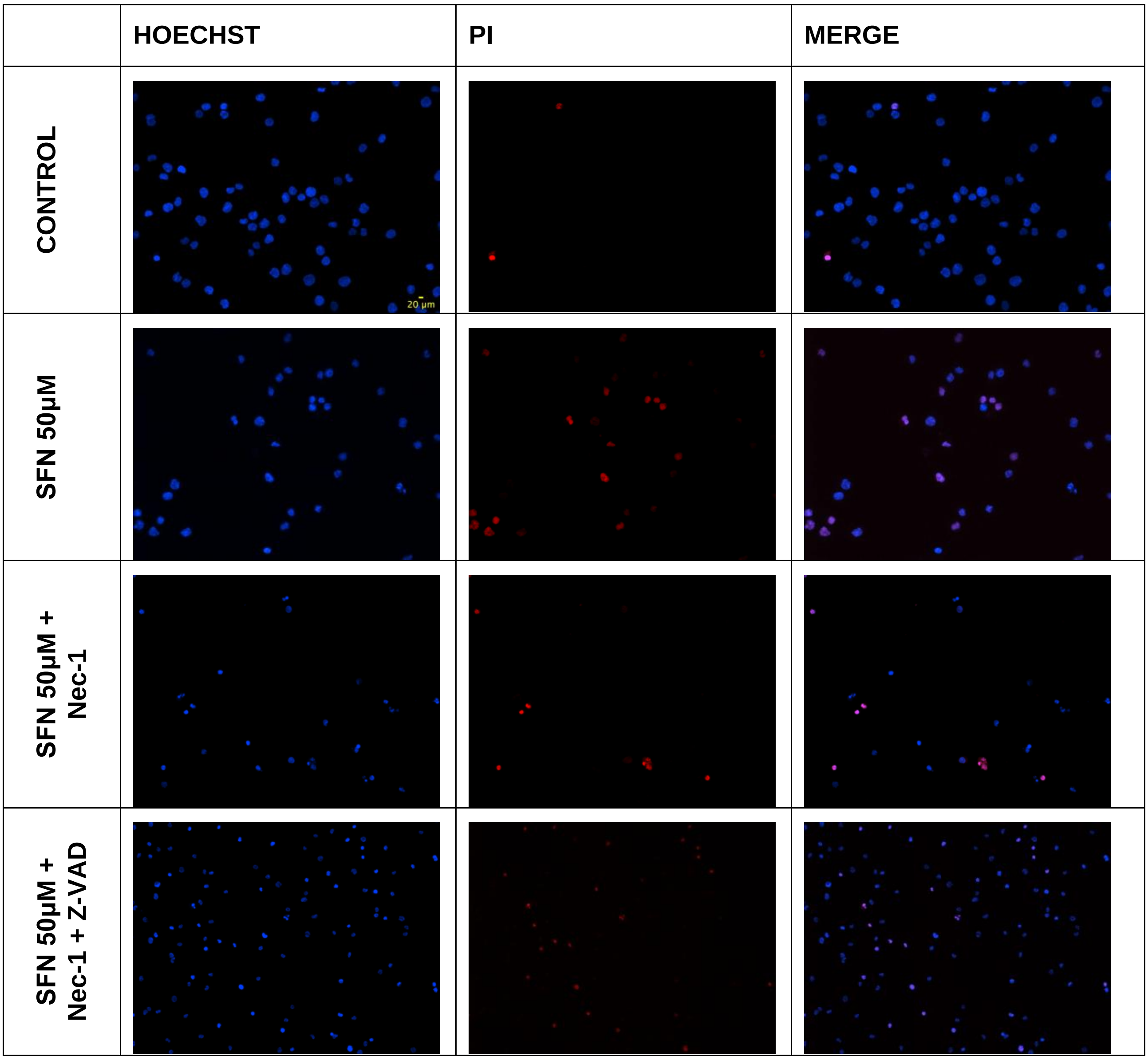

Figure S10. Representative images of the analysis of nuclear morphology by fluorescent microscopy of MV4-11 cells pre-treated with necrostatin-1 (Nec-1) with or without Z-VAD-FMK (Z-VAD) and then treated with SFN 50 µM for 24h.

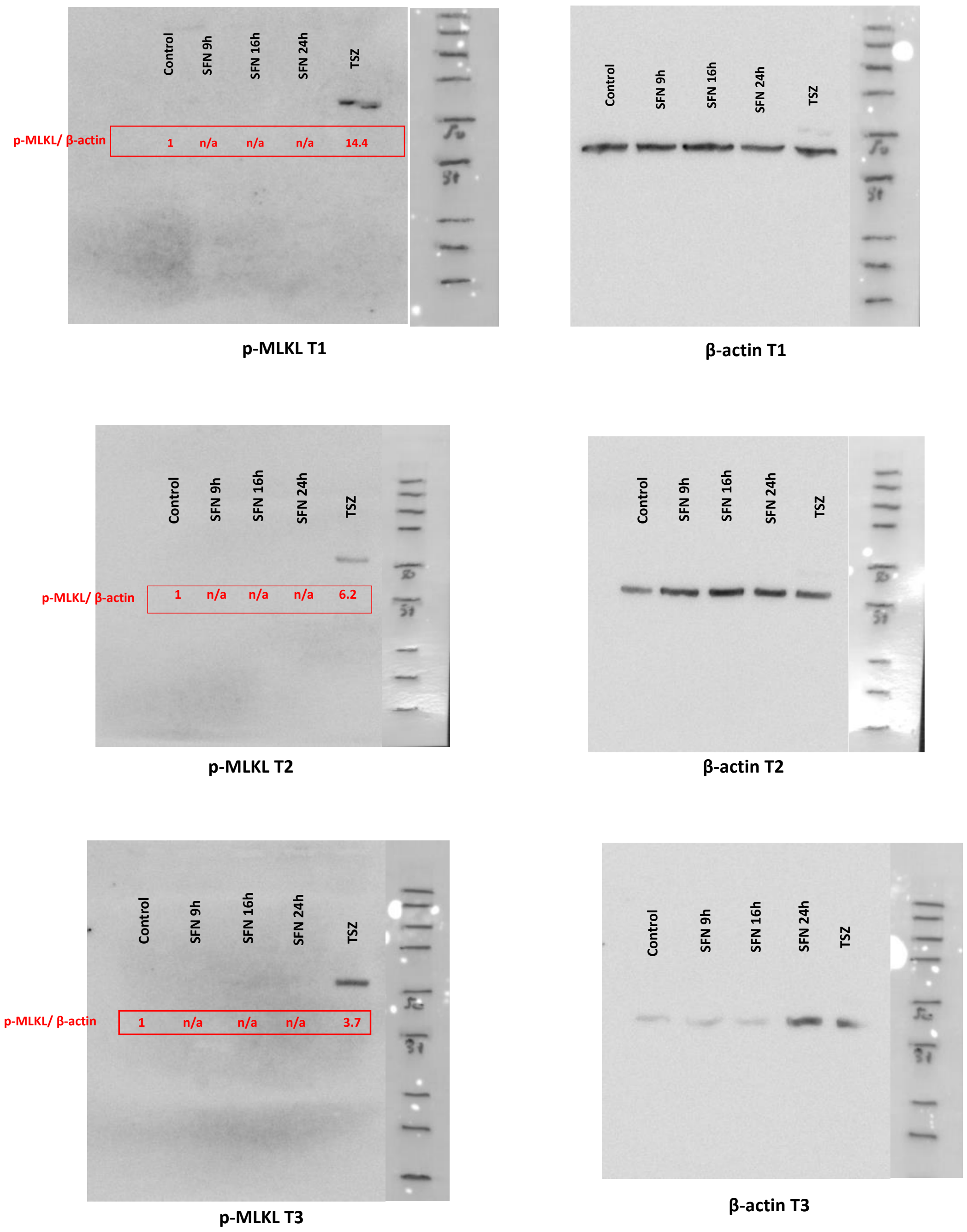

Figure S11. Uncropped images (whole Western Blot images) showing bands overlaid with the molecular weight marker of the three replicates related to the protein expression of phosphorylated MLKL (p-MLKL) and the respective protein expression of β-actin. Quantification is expressed as fold change *versus* untreated cells (normalized to β-actin).

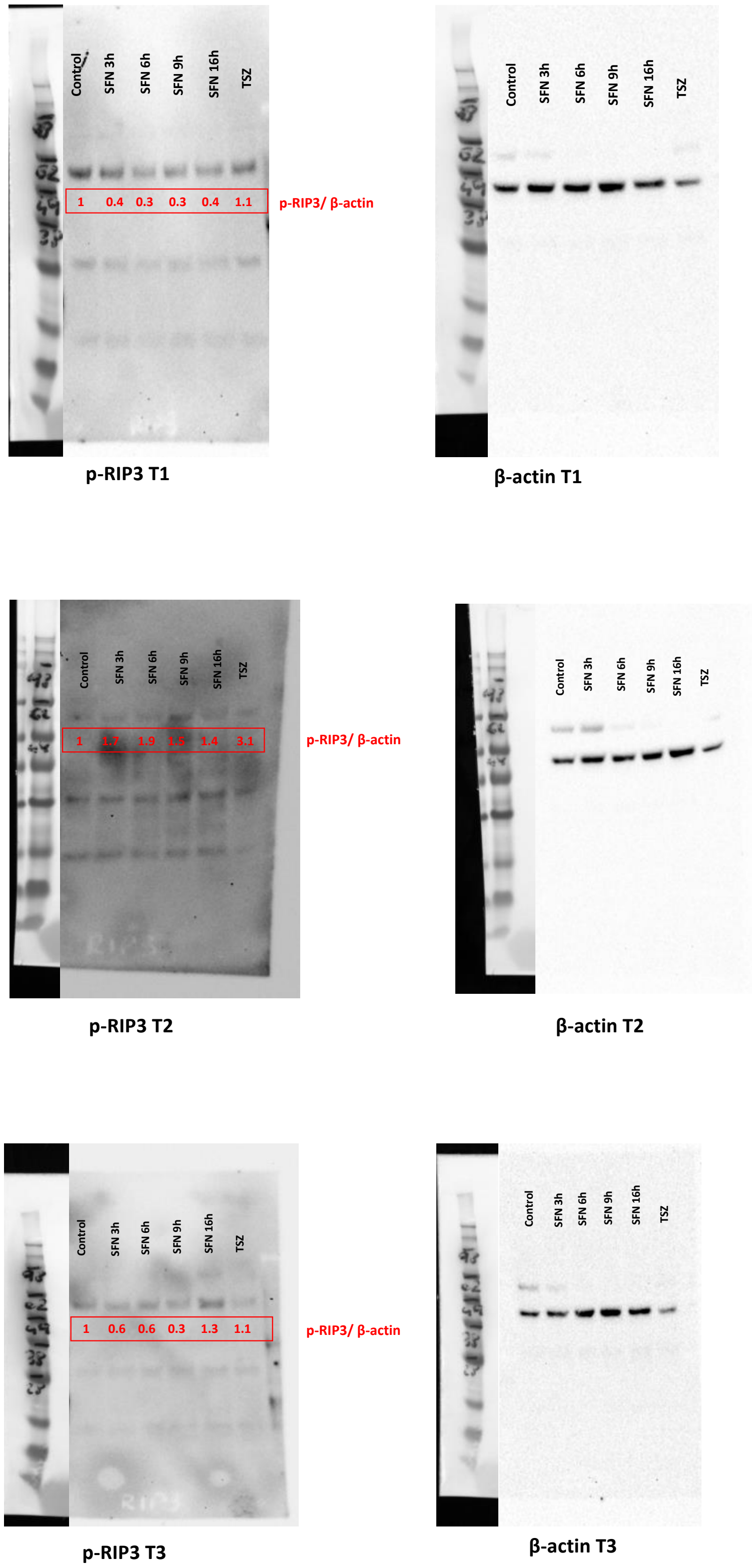

Figure S12. Uncropped images (whole Western Blot images) showing bands overlaid with the molecular weight marker of the three replicates related to the protein expression of phosphorylated RIP3 (p-RIP3) and respective protein expression of β-actin. Quantification is expressed as fold change *versus* untreated cells (normalized to β-actin).
